# Nanostructural Materials with Rare Earth Ions: Synthesis, Physicochemical Characterization, Modification and Applications

**DOI:** 10.3390/nano11071848

**Published:** 2021-07-16

**Authors:** Rafal J. Wiglusz

**Affiliations:** Institute of Low Temperature and Structure Research, PAS, Okolna 2, 50-422 Wroclaw, Poland; r.wiglusz@intibs.pl; Tel.: +48-071-395-42-74; Fax: +48-071-344-10-29

The success of nanotechnology in the field of physical, chemical and medical sciences has started revolutionizing the drug delivery science and theranostics (therapy and diagnostics) [1,2]. The specific advantages include superior pharmacodynamics, pharmacokinetics, reduced toxicity and improved targeting capability. This approach has great potential to produce novel diagnostics and therapeutics—theranostic—because of nanomaterials show unexpected and interesting chemical and physical properties different from those of the original in the micro-sized scale [3]. Therapies combining the use of bioactive materials and progenitor cells or an active substance become clinical reality, increasing the prospects for the development of engineering and regenerative medicine [4]. One of these perspectives is a diagnostics and personalized therapy, i.e., theranostics [5].

In this case of the active substance, the drug-delivery vehicle, as a critical quality attribute in the drug delivery science, needs special attention for the formulation development, which can be successfully achieved via nanotechnology. Drugs incorporated in nanocarriers, either physically entrapped or chemically tethered, have the potential to target the physiological zone of the disorder sparing normal cells from collateral consequences. Targeting several molecular mechanisms, for either treatment or prevention of difficult-to-treat diseases, for the design of various nanotechnology-based drug delivery systems is one of the prime focuses of the formulation scientist at the present juncture. 

Much attention has been devoted to developing new drug-delivery systems with many advantages compared with the conventional forms of dosage, such as, among others, enhanced bioavailability, greater efficiency, lower toxicity, controlled release [6,7,8,9,10,11,12]. An ideal drug-delivery system should be characterized by: (1) maximum biocompatibility and minimal antigenic properties [13]; (2) appropriate particle size, which is important for the particles to reach a particular location in the body due to the size of the vessels of the human circulatory system [14]; (3) the ability to transport the desired drug molecules to the targeted cells or tissues and release them in a controlled manner [15]. So far, different types of drug-delivery systems have been developed, such as, i.a. biodegradable polymers [16], xerogels [17], hydrogels [18], mesoporous materials [4,11]. Among different drug-delivery systems, mesoporous materials (such as SBA-15, MCM-41 and mesoporous silica nanoparticles) have gained increasing interest, particularly as drug storage and release hosts due to their unique surface and textural properties [14,19,20].

Materials designed for biomedical applications should be characterized by a high sensitivity and specificity, a lack of functional interference with the sample, photochemical stability, non-toxicity, long time of storage and, as far as possible, detection of a substance in the presence of others. Moreover, nanoscale materials have been exploited as active components in a wide range of technological applications in the biomedical field [21,22,23,24,25]. Particularly, in the field of biomedicine, nanoparticles can be used as drug-delivery vehicles that can target tissues or cells [13,24] and can be functionalized with special characteristics (such as magnetization, fluorescence and near-infrared absorption) for qualitative or quantitative detection of tumor cells [23,25,26,27].

It is well-known that nanoscale fluorescent materials have attracted much interest due to the increasing demand for efficient photosensitive materials not only for sophisticated optoelectronic and photonic devices but also for a broad range of biomedical applications [28,29,30,31,32,33,34]. In biomedical areas, luminescent materials, mainly including fluorescent organic molecules [35,36] and semiconductor nanoparticles [37,38], have been widely investigated in biological staining and diagnostics. However, some serious problems of photobleaching and quenching of fluorescent organic molecules and the toxicity of semiconductor quantum dots are critically evident and have seriously limited their applications in biomedical areas [38,39]. Furthermore, high performance in function-specific biological applications requires that the composites possess some unique characteristics, such as uniform morphology, large surface areas, good dispersion, etc. [39]. Recently, a class of stable, efficient and self-activated luminescent materials whose emission is induced by the defects or impurities in host lattices, has been prepared by various synthesis routes [40,41,42,43]. These novel self-activated inorganic materials may be a promising fluorescent material for biodetection due to their good optical properties and nontoxicity.

Apatites are inorganic compounds with a general formula M_10_(XO_4_)_6_Y_2_, where M represents divalent cations (e.g., Ca^2+^, Sr^2+^, etc.), XO_4_ = PO_4_^3−^, VO_4_^3−^, etc. and Y represents anions: F^−^, OH^−^, Cl^−^, Br^−^, etc. The hexagonal structure in apatites belongs to *P6_3_/m* space group and allows the cations to localize in the 4(f) and 6(h) positions [44] and is able to accommodate a variety of univalent cations as substituents. In that case, charge compensation, proposed by P. Martin and et al. [45], allows explaining the substitution of divalent calcium ions to trivalent lanthanide ions in apatite with a simple mechanism. It is worth mentioning that apatites themselves, such as calcium apatites Ca_10_(PO_4_)_6_(Y)_2_, are biocompatible and are natural building blocks for bones and teeth [46]. This feature combined with highly photostable luminescent properties of rare-earth dopants, makes nanocrystalline apatites highly attractive as luminescent bio-labels [47]. However, these materials have not been extensively synthesized or examined in the nanocrystalline form [48] which is a prerequisite for being internalized by cells for bio-imaging or sensing applications [49]. 

Several strategies have been developed in the synthesis of nanoparticles so far, involving such techniques as microemulsion, precipitation, thermal decomposition, chemical vapor deposition and others. However, the best results and control over particle size, crystallinity and purity can be ensured using microwave technology [50]. For instance, our group was able to obtain highly crystalline, phase pure, bio-compatible uniform and low agglomerated nano-apatites such as Ca_10_(PO_4_)_6_(OH)_2_ for bio-applications [51,52]. Another important feature is that the materials were produced in environmentally friendly conditions in ethylene glycol solution that is non-toxic for living organisms. Thus, this strategy seems to be very attractive for the synthesis of luminescent or multifunctional materials offering the possibility of bio-imaging measurement. The proposed synthesis technique allows for thorough control over the desired composition (as it was shown in the article [53]) which cannot be simply achieved using other techniques. Moreover, the proposed compounds can be considered as non-toxic due to their insolubility in body fluids and high chemical stability. It is well known that the solubility of oxide nanoparticles is one of the most important factors of their toxicity related to their chemical composition [54]. For example, the toxic effect of iron oxide nanoparticles originates mainly from the catalytic production of free radicals through Fenton type reaction [55]. To date, quantum dots (QD) characterized by high absorbance, high quantum yield, narrow emission bands and high resistance to photobleaching were considered as the most promising materials for FI applications in medicine. Currently, the main issue regarding QDs and their biomedical applications is their extreme toxicity (semiconductors—derivatives of highly toxic heavy metals such as Cd or Pb) [56]. One of the promising alternatives is offered by the application of inorganic compounds such as apatites doped or co-doped with optically active rare earth metals for bio-imaging [57]. 

Furthermore, calcium is the fifth most abundant element by mass in the human body (1.4–1.66%) where it is a common cellular ionic messenger with many functions and serves also as a structural element in bones (hydroxyapatites—99%) [58]. Calcium and its compounds play an important role in controlling numerous biological processes in living systems. Concentrations of free Ca^2+^ in biological cells are widely studied with fluorescent probes. The probes have a high selectivity for free calcium and exhibit marked changes in their photophysical properties upon binding. In particular, changes in fluorescence intensity (intensity probes) or spectral shift (ratio probes) upon binding to Ca^2+^ are monitored. The main drawback of intensity probes is that the intensity of fluorescence is affected by both the probe concentration and the free Ca^2+^ concentration. Consequently, a quantitative determination of Ca^2+^ distributions requires the probes to be distributed homogeneously in the sample. Conventional quantitative determinations of Ca^2+^ concentration with ratio probes overcomes the dependence on local probe concentration by exploiting ratiometric procedures using excitation or detection at two wavelengths [59,60]. The advent of fluorescence lifetime imaging techniques [1,61,62,63,64] opens new horizons for the quantitative determination for bio-imaging, in particular using intensity probes [65]. Fluorescence lifetime imaging is determined by factors such as the chemical environment of a fluorescent molecule and thus provides valuable information about its ion binding states. Importantly, since the lifetime is independent of fluorescence intensity, such measurements have wide-ranging applications to samples in which the probes have an inhomogeneous distribution. An additional advantage of the lifetime imaging technique is that the images are not compromised by photobleaching and absorption effects. 

Nanocrystalline probes doped with lanthanide ions based on apatites meet these requirements [66]. Their narrow emission lines as well as long life-times render them suitable for use as luminescent markers in biology and medicine [67]. Therefore, this strategy seems to be very attractive for the complete elimination of the effects associated with local concentration of ions in the sample. Moreover, the surface functionalization of nanomaterials with biologically active organic ligands results in a better stability of the colloidal dispersion. It will contribute to measurable progress in the possible extension of bio-imaging techniques. Independently of the scientific goal related to theranostics, the synthesis and study of spectroscopic properties of lanthanide-ion doped apatites could also be an important area of research.

## Data Availability

Not applicable.
